# Medicine and Longevity

**Published:** 1889-05

**Authors:** J. S. Todd

**Affiliations:** Atlanta, Ga., Professor Materia Medica and Therapeutics, Atlanta Medical College


### Address.

### PRESIDENT'S ADDRESS.

### MEDICINE AND LONGEVITY.

DELIVERED AT THE, FORTIETH ANNUAL SESSION, MEDICAL ASSOCIATION OF GEORGIA, BY J. S. TODD, M. D., ATLANTA, GA., PROFESSOR MATERIA MEDICA AND THERAPEUTICS, ATLANTA MEDICAL COLLEGE.

The paramount aim of medical science is to prolong human life, and hardly less to mitigate human suffering.

I propose in this address to show, that from the beginning the great body of professional men have been true to the claims of their high calling.

Thirty and three years is usually regarded as the average age of man, and this conviction is so universal that he must indeed be a bold man who questions its exact truth. It was with some trepidation, therefore, that I began to investigate this matter of longevity and its relations to medicine.

I determined that, if after a fair investigation, I found this axiom to be true, I would spare no pains to discover the cause of this strange and somewhat humiliating failure. A failure, certainly not the less humiliating because exceptional in the brotherhood of the learned professions.

Our legal brethren have greatly benefitted mankind by improved legislation and by wise judicial administration. Why should the sons of Esculapius be less successful than the disciples of Themis ?

Again, the clergy, by their teachings, have contributed vastly to the betterment of public and private morals. Why should medical men be laggards in this work of upbuilding our common humanity?

Were not medicine-man and priest or prophet convertible terms in the earlier ages? Is not Christianity itself a Divine therapeutics? And ought not Medicine in the future, as in the past, be the handmaid of Religion?

As one result of my investigation, I proclaim and stand ready to defend this proposition, that of all avocations the scientific members of our calling are surely the meekest and most long suffering.

When we consider the matter-of-course way in which fun is poked at us by the press, the pulpit, the bar and the stage, and our good humored acquiesence in it all, I think I will be guilty of no irreverence if I announce that I believe when St. Paul said, "Charity suffereth long, and is kind, vaunteth not itself, is not puffed up, seeketh not her own, is not easily provoked, beareth all things," he was with prophetic eye describing the regular doctors of this age.

As a striking illustration of the humility and unpretentiousness of representative men in our profession who make themselves absurd, I was once severely taken to task for having innocently remarked that I had cured a patient of a certain disease. Said this Caiaphas in the "medical temple:" "We cure nothing. I know of no ailment that is cured. Many diseases run their course and the patient survives, often gets well despite the treatment."

Now just such extravagant assertions as those just quoted have given to the outside world a clew, something used by them, as by detectives, for defaming the innocent, making allegations which can not be substantiated.

An editorial paragraph of a leading daily in this State about a year ago read thus: "In Russia they have but one doctor to 20,000 inhabitants, and yet the people are as long-lived as in less favored (?) climes." Now this half humorous fling at the medical men was penned through ignorance, but the inspiration of the article was from just such remarks as that made by this learned M. D., who "cured nothing, and did not believe in drugs."

I am indebted to this secular editor for my subject ; for then and there, I began to think about the necessity for information on longevity, etc.

Webster defines cure, medically, to mean "to heal, to restore to health, soundness or sanity ; to subdue or remove by remedial means ; to bring relief of, to remedy, to remove."

Is it an optical illusion that I labor under when, after a course of medicine, in the place of foul ulcers, healthy integument gladdens my eyes ?

Was there a packed jury, a conspiracy, always among the panel that said this man who, after treatment, again sits on the bench, fills the pulpit, or wields the scalpel, was once crazy ?

Does my thermometer lie when it tells me that after I have given anti-piretics the temperature is lowered ?

Is he still in darkness when a dexterous hand has removed from his eye a cataract, and he welcomes with glad shouts the advent of light ?

Is it possible our patient is deceived when he thanks us for that hypodermic of morphine, and says "I have pain no longer?"

Do all men fain sleep after a hypnotic potion ?

Must we come to the conclusion that, after all, misrepresentation on the part of our patrons has bewildered us, and, despite the fact that quinine has been administered, they still shake, their assertions to the contrary notwithstanding ?

No, no, what if sulphur was applied for his tormenting itch, he has pruritis yet, only the innate tendency to prevaricate prompts him to deny it.

How would this sound ? Before the tendon of Achilles was cut, the patient with club foot could have put his heel flat on the ground ; now, being divided, you see he does it, but the tenotomy had nothing to do with it.

The doctor who says he never cured an ailment may be telling the truth, doubtless is, for his clientage will surely grow less, whether from their disgust or early demise. I know not which—

but he speaks for himself—he is not authorized to affirm for this body, I opine, *certainly not for the witness now on the stand*.

From Kropotkin, a Russian statistician, I learn that "the mortality in Russia is greater than anywhere else in Europe, the lowest figures are found in Courland, 20 per 1000; Baltic province, 22 and Poland, 30." In the first two provinces M. D.'s are as plenty as in Germany. "Within Russia itself the rate varies between 29 and 49 (and 30 to 38 in cities)."

"In 1882 the average mortality in the 13 central governments reached the exceptional figures of 62 per 1000."

"The mortality is highest among children—only one-half of those born reaching their seventh year."

"From military registers it appears that of 1000 males born, only 480 to 490 reach the twenty-first year, and of these only 375 are able-bodied. Of the remainder, who are unfit for military service, 50 per cent suffer from chronic diseases."

"Misery, insanitary dwellings and want of proper food, account for this high mortality, *which is further increased by the want of medical help*, there being, in Russia with Poland, only 15,348 male and 66 female surgeons and 7679 assistants."

"In sixty-three governments, having an aggregate population of 76,000,000, there were only two surgeons to 100,000 inhabitants."

It is worthy of note that in the United States, where there is one doctor to about every 600 people, we have the least mortality.

England leads next in health, and in the number of M. D.'s, with one physician to 1500 inhabitants, and Russia, with her ghastly death rate, has, of all enlightened people, fewer medical men to total population.

Is it not possible, in view of these figures, that a multitude of Esculapians are not such an unmitigated evil as many of us even have thought? In Odessa, in Russia, 40 per cent of the whole population and 94 per cent of the very poor die without having had medical advice.

The people surely are not the sufferers because of our muchness; and the plan of outliving disease without treatment does not (to use a mining expression) "pan out" when put to the actual test.

In Anam, where there is no real medicine, the average of life is now 26.5 years to a generation.

With these preliminary remarks, I ask your close attention to some statistics, which I have, with much labor and some expense, succeeded in getting together.

The true era of statistics is just beginning, and owing to the absence of boards of health, there is still just a start in the United States.

My efforts, arduous as they have been, have already been requited by the gratification which they have afforded me ; but my cup will be filled to overflowing if the exhibit that I make will convince the people that we are up and doing, that we have lengthened the span of life ; that we do alleviate, yes, *cure;* more than this, we have banished many of the scourges of mankind ; and that we will and can, if they *accord us the power*, procure for them more happiness, greater freedom from suffering, and longer life.

Says Dr. Gihon, of the United States Navy, who has contributed much time and added so largely to our scant stock of knowledge on medical demography : "This much we know definitely, that the general death rate is diminishing, and the average length of human life and the duration of a generation on the earth proportionately increasing, there is less sickness per capita than when observers began to keep a record of such matters."

M. Korosi, (in 9th transactions Inter-Md. Congress) the statistician of Buda-Pesth, has come to the conclusion that in the human family, among the wealthy class, they reach the average age of fifty-two, the middle class forty-seven, and the poor rather more than forty-one and a half, which gives a general average of about forty-six years.

"It is estimated," says Dr. Felton, the learned Georgia statesman, divine and M. D., in an address before the graduating class of Atlanta Medical College, "that human life has increased twenty-five per cent in the last fifty years."

The average human life in Rome, under Cæsar, was eighteen years, now it is forty.

The average in France fifty years ago, was twenty-eight; the mean duration in 1887 was forty-five and a half years.

In Geneva, during the thirteenth century, a generation played its part upon the stage and disappeared in fourteen years ; now the drama requires forty years before the curtain falls.

During the golden reign of good Queen Bess, in London, and all the large cities of merry old England, 50 out of every 1,000 paid the last debt to nature yearly; which means that, instead of three score and ten, they averaged but one score *Now, in the city of London, the average is forty-seven years.*

For the following table I am indebted in part to Dr. Billings, of Washington, who says it permits of as near a probable comparison of the annual death rate in the various countries named as can be procured:

| COUNTRY. | Period | Death rate per 1,000 living pop. | Life average expectancy. |
|---|---|---|---|
| United States | 1879 and '80 | 18. | 55 years |
| England | 1880 | 20.5 | 50 |
| England, rural districts | 1880 | 18.5 | 54 plus |
| Denmark | 1880 | 20.4 | 50 |
| Sweden | 1880 | 18.1 | 55 nearly |
| Austria | 1880 | 29.1 | 34.5 |
| German empire | 1880 | 26.1 | 38.7 |
| Italy | 1880 | 30.5 | 33.7 |
| Belgium | 1880 | 22.4 | 45.5 |
| France | 1860 to '77 | 23.6 | 42.5 |
| Spain | 1861 to '70 | 29.7 | 34 minus |
| Russian empire | 1876 to '82 | 36. | 27.8 about |
| Chili | 1876 | 36. | 27.8 about |

The following table, kindly sent me by Dr. J. S. Billings, will show that in the countries named the death rate is gradually decreasing:

DEATHS PER 1,000 OF POPULATION.

| German Empire. | France. | Prussia. | Belgium. | Netherlands. |
|---|---|---|---|---|
| 1880, 26.0 | 1860 to '77, 23.6 | 1886, 26. | 1880, 22.4 | 1886, 21.8 |
| 1887, 24.2 | '87, 22. | '87, 23.9 | 1887, 19.3 | 1887, 18.7 |

He says in the letter inclosing the table: "The mortality rate is lower in all civilized countries than it was in 1880."

In contrast to the above it is stated by a recent writer that in Ellobed, the capital of the Soudan, 23 years is the average life of its citizens.

But we need not go so far from home to see what prompt and efficient medication will do.

The following tables give the life expectancy of white and colored inhabitants in the cities and places named.

CENSUS 1880

| | At birth, expectancy. | After five years will live to be. |
|---|---|---|
| District of Columbia: | | |
| White | 42 years. | 57 years. |
| Colored | 25 " | 47 " |
| Baltimore: | | |
| White | 38 " | 57 " |
| Colored | 23 " | 49 " |
| Charleston: | | |
| White | 38 " | 53 " |
| Colored | 21 " | 45 " |
| New Orleans: | | |
| White | 38 " | 52 " |
| Colored | 37 " | 42 " |

The figures speak for themselves; comment is useless.

Lest I mislead when I use the expression "life expectancy," let me explain. For instance, you will find, in all probability, as many old people in Germany as in France, but the greater infant mortality in the former country lessens the general average. The greater death rate during the first years of life in Prussia, over France, is due to the fact that the average occupants of a house in Berlin is 57, in Paris 29, (in London 8,) showing the fatality to children of overcrowding. The expectancy table will, of course, vary, as will the death rate, but is a fair criterion, and on the whole may be relied on. As an illustration of the reliability, the average death rate in all England was for the period 1841 to 1876—35 years—21 per thousand, and there has been a steady increase in healthfulness since the latter date.

To Mr. Vacher we are under obligations for the following comparative table. Sweden has an uninterrupted statistical record since 1740.

| Country or City. | Period. | Death rate per 1,000 living population. | Life average expectancy. |
|---|---|---|---|
| Sweden | 1770 to 1790 | 28.5 | 35. about. |
| Sweden | 1880 to 1885 | 17.5 | 57. " |
| Copenhagen | 1770 | 39. | 26. " |
| Copenhagen | 1880 to 1885 | 23.2 | 43. " |
| Berlin | 1720 to 1724 | 41.0 | 24.5 " |
| Berlin | 1880 to 1885 | 26.6 | 38.5 " |
| Duchy of Milan | 1774 | 41.3 | 24. " |
| Duchy of Milan | 1885 | 27.9 | 36. " |
| Rome | 1794 to 1800 | 39.0 | 26. " |
| Rome | 1880 to 1885 | 26.7 | 37. " |
| France | 1800 | 34.0 | 38. " |
| France | 1887 | 21.00 | 47½ " |

According to the statistical and official annual reports of the city and suburbs, there died of each 1,000 inhabitants of Vienna, irrespective, whether transient, or resident, or brought from the exterior into the hospitals there :

1853-67 .......... 38.0

'67-70 .......... 28.8

'71 } (typhoid fever epidemic) { .......... 32.5

'72 } .......... 34.3

'73 .......... 23.8

'74 .......... 26.6

'75 .......... 26.6

'76 (cholera year) .......... 28.2

'77 .......... 26.5

'79 .......... 25.9

'80 .......... 24.0

'81 .......... 23.4

'82 .......... 25.3

'83 .......... 24.3

'84 .......... 23.3

'85 .......... 24.8

'86 .......... 22.3

The great increase in '71 and '72 was due to the bad condition of the city's drinking water.

In Ireiginia, a small country town of 2,800 people, in France, Mr. Vacher found that from 1775 to 1790, the death rate was 41.4 per 1,000 inhabitants and the average of life 23.6 years. These averages are now 24.3 and 35.9 respectively.

Five of the oldest life insurance companies in America, chartered in 1835-42-43-45 and '46 respectively, averaging forty-five years old, and representing nearly 400,000 lives, report that the actual to expected deaths have been about 85 per cent ; and this on the American and not the combined experience tables of Europe and America which calculated on a *much greater* mortality than the tables now used by them.

More conclusive proof of the lengthening days of man upon the earth could not possibly be obtained. Life insurance was practiced in England during the reign of Queen Elizabeth, and has been reduced almost to a science ; it is *money* evidence in favor of my position.

The tremendous accumulations running up in single companies to the hundred million and over, despite the average twenty-five per cent annual dividend, or return of money overcharged the insured, tells undeniably that their calculations, even now, are based on shorter-lived people than live in civilized countries.

Now, I do not wish to be understood as claiming that the doctors, and they alone, are to receive all the credit for this favorable showing ; but, with Doctor Felton, I agree, when he says : "From the day that Hygeia, the daughter of Esculapius, was born, till this, every idea of the present time, valuable to the world for the prevention of disease, has been originated by the medical profession ; civil engineers and civil authorities have only been the executive force of this imperial power behind the throne."

Drainage was the outcome of the deliberations of a board of physicians, under the patronage of the Royal Society of London, then in its infancy, that has banished the plague from Europe.

We propose to review a few of the factors that medicine has directly and incontestably contributed to lengthen human life and mitigate suffering.

It is justly claimed that Jenner, by the discovery of vaccination, added three years to our span, to say nothing of the freedom from the indescribable agony that this loathsome disease occasions.

In the wilds of the New World there was discovered a tree whose bark has been used for the healing of the nations—*cinchona.*

Malaria, of *all* causes of disease, is the most wide-spread. There are *few* places where it does not exist and implant its influence on the malady, even when not the sole cause of the sickness.

It has been no respecter of persons or any age, for Cæsar had a fever when in Spain—

> "And when the fit was on him I did mark
> How he did shake ; 'tis true, this god did shake."

says the envious Cassius.

James I., of England, and Cromwell were victims of its malignity. We antidote malaria so certainly with the salts of cinchona now, that it sounds odd to use the word malignancy in connection.

I believe cinchona has added at least a couple of years to the average age of civilized men—how many years to his usefulness while living it would be hardly possible to overestimate.

The complete control we have over pain, afforded by its masters, *opium and the anesthetics*, not only saves suffering, but life itself.

Before the days of opium, (and it was first used as late as about the middle of the fifteenth century by the Arabs) who, but God, knows how many died who would have been saved had its virtues been known then as now ? It is one of the blessings of our time that, like air and water, we do not sufficiently appreciate.

Pain is competent, uncombatted, to cause death itself. Sleep is impossible when pain is present ; without sleep existence rapidly draws to a close ; but, before this merciful termination, insanity or lockjaw is apt to occur.

The continuance of pain in any organ, sooner or later, leads to a determination of blood to that part, for "*Ubi irritatio ibi affluxus*," and this is but the beginning of an inflammation which has, as one of its results, not only a destruction of the part, but of the individual. Therefore, opium, checkmating the first herald of many inflammations, is one of our best preventive or abortive agents in combatting disease.

But the many virtues of this greatest gift of God to man would require a volume to enumerate.

If it had no virtue but that of producing euthanasia—a painless exit to the beyond—it would still be an inestimable boon.

There comes a time when all hope is abandoned; the messenger on the white horse is at the door and demands a victim; our patient's feet already in the icy Styx; the cold dew of death upon his brow;

"When the life of all his soul
Touched corruptibly, and his pure brain,
Which some suppose is life's frail dwelling-house,
Doth by the idle comments that it makes
Foretell the ending of mortality;"

Even then, thank God, there is "balm in Gilead:"

"For when, O Death, thou wouldst carouse,
Bind the poppy round thy brows.
Bind it round with bonny glee,
'Tis the fittest wreath, Oh! Death for thee."

Mercury is to medicine what Homer is to verse:

"That bard sublime,
Whose distant footsteps echo
Through the corridors of time;"

the Sampson of the drug-store, the two-edged sword, the *universal stimulant* to excretory and secretory glands, and is capable of entering the blood, grappling with disease and coming off the victor; nay, more, meets it at the very threshold and bars its entrance! Fearlessly may Dr. Billings write that the death rate is lower in all countries in 1889 than in 1880, for since that time the teachings of Lister have made themselves felt, and practiced wherever scientific medicine exists!

Antisepcis—a term that is almost synonymous with corrosive sublimate—means more than mere cleanliness; but if we were forced to accept the dogma that it was cleanliness simply, still it would be next to godliness, has banished surgical and puerperal fevers, and made possible operations that were not dreamed of before its introduction. Wisely have the German law makers made it illegal for surgical operations to be performed without practicing it.

Syphilis, once the scourge of Europe, has, I believe, had its virus so attenuated through rational treatment by this drug, that it is no longer the dreadful and loathsome malady that it was in former times.

Mercury is rapidly doing for society, in the mitigation of this plague, that which for moral reasons the law *never would* have tolerated.

Where medicine in its true sense—as practiced among us—is unknown, let us see what ravages syphilis is making. In Brazil, sixty per cent. of the infant mortality is attributed to hereditary syphilis. In Corea, syphilis is general. In Tahiti, it is less common than formally, but still the national curse. In Japan, syphilis of the most violent type prevails as fatal *as among the black races.* In Hawaii, the destruction of the aborigines has been hastened by syphilis and smallpox. Veneral diseases in every form are among the chief curses of Bellany, Hyderabad and the Nizams' dominions in India; in these doctorless corners of the earth, it is seen in its most severe forms, it has the most disasterous effects on the health of the *reputable class of women*, thirty per cent. of whom are affected. In Mexico and Chili, it is everywhere prominent, undermining and sapping the national strength. Tens of thousands of Chinese die yearly from its venom,—but I forbear.

With efficient boards of health, cholera would, with plague, be confined to its birth-place, in the marshes of its Chaldee home. Small-pox in China, *to-day seen everywhere*, would become a hideous memory of the past. During the census year, there died in the United States 756,593 persons; 100,000 of diseases which were in a sense, Dr. Billings declares, unnecessary or preventable—nearly one-seventh of the annual mortality needless!

I am persuaded that the germ theory of contagious and infectious diseases will give us, in the near future, immunity from typhoid fever, diptheria, and many other diseases, by as simple a formula as that which drove away the plague from Europe, which was drainage. It is this, pure water (not chemically pure necessarily,) for it may be chemically impure, "reeking," says Huxley, and still be drank with impunity. On the other hand, water chemically pure, sparkling, inodorous and tasteless, may yet have in it germs, which while not so instantaneously fatal as prussic acid, are equally as deadly." Pure water may be chemically condensed, or not pleasant to the taste or olfactories—as, for example, artesian water—yet healthy to drink; on the other hand, approved by the chemists, but poisonous.

Therefore, when there is an epidemic of cholera or typhoid fever, stirilize the water by boiling before it is imbibed, kill the germs of infection with the great purifier—heat.

The typhoid germs were found in the water of the Ohio river when typhoid fever was so prevalent there a year ago, and also in the waterworks, and later in the wells in Wilkesbarre, Pa., during their fearful epidemic, two years since, of this disease.

Yellow fever should and can be kept from our shores, for it has existed in Quebec, and was for years the scourge of Philadelphia. But boards of health have driven it from New England, the middle and north Atlantic States, to the southern seaboard *and it will continue to occur there until we are authorized by the law makers to expel it.*

To prolong human life and prevent disability one day in the United States, granting that the daily earning capacity is one-fourth of $1 capita, means a saving of $12,500,000.

Think of the difference between one day, and thirteen years, and you will have an idea of the part we have had in making this era, the richest and best ever known in the world's history!

I reckon on the basis of thirty-three years to a generation one hundred years ago, and forty-six now, which, for civilized countries, is not too high. The figures, if made, would be so innumerable that they would bewilder.

When Augustus Cæsar died, he boasted that posterity would say of him that he found Rome brick but left it marble.

The *underground* London of to-day involved more human labor and cost more gold than all that was of the Eternal City did then, when she was at the zenith of her power, her glory and her wealth!

I am firmly persuaded that if the State of Georgia would have published, and send free, a weekly journal of health to every voter in her boundaries, she would make an investment that would give her more wealth, and in a short time draw more population to her borders than any single thing she could do.

Such a journal edited by say Dr. Eugene Foster, of Augusta, would, in a short while, so enthuse and interest the people about sanitary measures, that the popular clamor for a board of health would be irresistible. The cost of such a publication would be trival, when we consider the advertisements that would seek entrance into a journal with over three hundred thousand circulation.

Suppose all the lamps in the chemical laboratories were extinguished; close up all the drug-stores after emptying their contents in the streets, literally "give physic to the dogs;" destroy all surgical, gynecological and opthalmic instruments, and orthropedic appliances; fancy that all recollection of the remedial powers of things on earth, in air and water was a blank—make fabulous all we have learned of anatomy and physiology, and does any sane man believe that the generation to come would be as vigorous, as healthy, as long-lived or as happy as this one?

Right here, after thorough investigation, let me authorize you to say that the prevalent idea that parturition among nomads or savages is purely a physiological and never fatal act is a *great mistake*. From the time that Rachel travailed with Benjamin and *died* in the way to Ephrath, down to this day, the mortality is greatest, *even in giving birth*, where the ideas of medicine are the most crude. A placenta previa, or position that requires turning, with them means *death*.

Finally, gentlemen, after thanking you for the unmerited honor you have conferred upon me, by making me your president, pardon me for saying that you could not have selected one of your number who was more in love with our profession in all its department and details.

I love and venerate my avocation, singly,—as a body because it commits to my care *man*, whom God made a little lower than the angels—*human life*, the most precious of all created things—flesh and blood that is the birth-place of the soul, the mother of the immortal.

I am happy in practicing it, for it acquaints me with the hidden things of the earth, that He, who first loved us, had stored away from the beginning in Nature's womb, to be delivered to the diligent searcher. I am glad I am a disciple of Esculapius, for its teachings comport with those of Revelation, which, summed up, read, "To live, to be happy—walk in the paths of virtue pursuing truth."

Feeling that I have been rewarded, even when no fee is received after relieving pain; knowing that the followers of Galen regard all men as their neighbors when in distress, what have I to make me ashamed or afraid?

If every moment of life is a monument of mercy, I rejoice with an exceeding great joy, confident that we have, by a right use of the agents made for us, *lengthened* man's stay on earth. And I rejoice the more when I feel assured there waits for all of us, who, with God's help, have "served their generation" here on earth, when earth is past, an "abundant entrance" into that City, where the inhabitant shall not say "I am sick," where there is no death, but rather

"*Life* in fullest glow
And where the light is golden,
And milk and honey flow;
The light that hath *no evening*,
*The health that hath no sore*,
The life that hath no ending,
*But lasteth evermore*."

